# Diagnostic accuracy of deep learning in detection and prognostication of renal cell carcinoma: a systematic review and meta-analysis

**DOI:** 10.3389/fmed.2024.1447057

**Published:** 2024-09-05

**Authors:** Deepak Chandramohan, Hari Naga Garapati, Udit Nangia, Prathap K. Simhadri, Boney Lapsiwala, Nihar K. Jena, Prabhat Singh

**Affiliations:** ^1^Department of Nephrology, The University of Alabama at Birmingham, Birmingham, AL, United States; ^2^Department of Nephrology, Baptist Medical Center South, Montgomery, AL, United States; ^3^Department of Medicine, University Hospital Parma Medical Center, Parma, OH, United States; ^4^Department of Internal Medicine, Medical City Arlington, Arlington, TX, United States; ^5^Department of Cardiology, Trinity Health Oakland Hospital, Pontiac, MI, United States; ^6^Department of Nephrology, Christus Spohn Health System, Corpus Christi, TX, United States

**Keywords:** machine learning, deep neural network, pathomics, renal cell carcinma, artificial inteligence-AI

## Abstract

**Introduction:**

The prevalence of Renal cell carcinoma (RCC) is increasing among adults. Histopathologic samples obtained after surgical resection or from biopsies of a renal mass require subtype classification for diagnosis, prognosis, and to determine surveillance. Deep learning in artificial intelligence (AI) and pathomics are rapidly advancing, leading to numerous applications such as histopathological diagnosis. In our meta-analysis, we assessed the pooled diagnostic performances of deep neural network (DNN) frameworks in detecting RCC subtypes and to predicting survival.

**Methods:**

A systematic search was done in PubMed, Google Scholar, Embase, and Scopus from inception to November 2023. The random effects model was used to calculate the pooled percentages, mean, and 95% confidence interval. Accuracy was defined as the number of cases identified by AI out of the total number of cases, i.e. (True Positive + True Negative)/(True Positive + True Negative + False Positive + False Negative). The heterogeneity between study-specific estimates was assessed by the *I*^2^ statistic. The Preferred Reporting Items for Systematic Reviews and Meta-Analyses (PRISMA) guidelines were used to conduct and report the analysis.

**Results:**

The search retrieved 347 studies; 13 retrospective studies evaluating 5340 patients were included in the final analysis. The pooled performance of the DNN was as follows: accuracy 92.3% (95% CI: 85.8–95.9; *I*^2^ = 98.3%), sensitivity 97.5% (95% CI: 83.2–99.7; *I*^2^ = 92%), specificity 89.2% (95% CI: 29.9–99.4; *I*^2^ = 99.6%) and area under the curve 0.91 (95% CI: 0.85–0.97.3; *I*^2^ = 99.6%). Specifically, their accuracy in RCC subtype detection was 93.5% (95% CI: 88.7–96.3; *I*^2^ = 92%), and the accuracy in survival analysis prediction was 81% (95% CI: 67.8–89.6; *I*^2^ = 94.4%).

**Discussion:**

The DNN showed excellent pooled diagnostic accuracy rates to classify RCC into subtypes and grade them for prognostic purposes. Further studies are required to establish generalizability and validate these findings on a larger scale.

## 1 Introduction

Renal Cell Carcinoma (RCC) is the most common primary renal neoplasm, affecting nearly 300,000 individuals worldwide annually, and it is responsible for more than 100,000 deaths each year (1). RCC is a heterogeneous group of cancers with distinctive molecular characteristics, histology, clinical outcomes, and therapy response. RCC arises from the renal parenchyma and, according to the World Health Organization (WHO) has three main subtypes: Clear cell (ccRCC), Papillary RCC (pRCC) and Chromophobe. The remaining subtypes are rare, each occurring with a total incidence of ≤1%. Each type has different histologic features, distinctive genetic and molecular alterations, clinical courses, and different responses to therapy (2).

The ccRCC type accounts for 70–90%. It is named due to the presence of clear cells from the lipid and glycogen-rich cytoplasmic content, ccRCC has the worst prognosis among the RCC subtypes with a 5-year survival rate between 50 and 69%. When metastasis occurs, the 5-year survival decreases further to about 10%. The pRCC type has a spindle-shaped pattern of cells with areas of hemorrhage and cysts. Pathologists further classify it into two subtypes based on the lesion’s histological appearance and biological behavior, and it accounts for about 14–17% of the cases. The subtypes, pRCC type 1 (basophilic) and pRCC type 2 (eosinophilic) differ in their prognostic significance, with type 2 having a poorer prognosis. Chromophobe RCC is common in adults over the age of 60 years. Histologically described as a mass formed of large pale cells with reticulated cytoplasm and perinuclear halos, it carries the best prognosis among the RCC types in the absence of sarcomatoid changes. If sarcomatoid transformation occurs, it tends to be more aggressive with worse survival (3).

Due to its relevance and applicability, the Fuhrman nuclear grading method is commonly used for staging to determine prognostic significance. Using nuclear morphology and characteristics, it designates a prognostic indicator grade (4). The histological classification of RCC is of great importance in patient care, as RCC subtypes have significant implications in the prognosis and treatment of renal tumors. The incidence of RCC has increased, likely due to the increased detection of incidental renal masses on abdominal imaging (5). Around 60% of RCCs are detected incidentally (6). The inspection of complex RCC histologic patterns is prolonged and time consuming due to tumor heterogeneity. There is also a moderate amount of inter-observer and intra-observer variability due to the absence of a defined threshold for determining the minimum percentage of an area with high nuclear grade (7).

With the advancement of whole-slide images in digital pathology, automated histopathologic image analysis systems have shown great potential for diagnostic purposes (8–10). Computerized image analysis has the advantage of providing a more efficient, less subjective, and consistent diagnostic methodology to assist pathologists in their medical decision-making processes. In recent years, significant advancement has been made in understanding and applying deep neural network (DNN) frameworks, especially convolutional neural networks (CNNs), to a wide range of biomedical imaging analysis applications. These CNN-based models can process digitized histopathology images and learn to diagnose cellular patterns associated with tumors (11, 12). In our systematic review and meta-analysis, we provide a comprehensive assessment of the existing literature and present the pooled diagnostic performances of DNN frameworks in detecting RCC and predicting outcomes.

## 2 Materials and methods

### 2.1 Data sources and search strategy

The literature search was conducted from inception through December 2023 in the following electronic databases, Pubmed, Embase, Web of Science, Cochrane Library, and Google Scholar, using the following terms, “Renal Cell Carcinoma” OR “RCC” OR “Kidney Cancer” AND “Histopathology” OR “Histological Analysis” OR “Tissue Histopathology” AND “Deep Neural Network” OR “DNN” OR “Deep Learning.” Additional pertinent studies were added by searching the bibliographic section of the articles of interest. The search strategy is shown in the Supplementary data section.

### 2.2 Study selection

The studies retrieved from the search were screened by two authors (D.C and P.S). Abstracts of the studies were initially screened, followed by full-text screening to include studies based on prespecified inclusion and exclusion criteria. Any disagreements between authors were resolved through consensus. The Checklist for critical Appraisal and data extraction for systematic Reviews of prediction Modelling Studies (CHARMS) for prediction modeling studies was followed (13) and The Preferred Reporting Items for Systematic Reviews and Meta-Analyses guidelines was used to select the final articles (14). The CHARMS and PRISMA checklists are shown in the Supplementary data section. The study protocol was registered in PROSPERO, a database of systematic reviews, with registration number CRD42024497980.

The inclusion criteria were as follows: (1) studies reporting the histopathological diagnosis of RCC using DNN; (2) studies reporting detection of RCC using DNN models after validation. The exclusion criteria were as follows: (1) studies lacking sufficient data on reported accuracy, sensitivity, specificity, positive predictive value, negative predictive value or area under the curve of DNN models; (2) review articles, conference abstracts and case reports; (3) studies conducted on animal models; (4) studies not published in English; (5) studies reporting data on DNN models predicting RCC based on imaging; (6) studies reporting only the mathematical development of DNN models without internal or external validation and (7) studies that reported RCC detection using methods other than DNN. Ethics approval was not required for our meta-analysis because the data was accessible to the public.

### 2.3 Outcomes assessed

The outcomes assessed were accuracy, sensitivity, specificity, and area under the curve (AUC) of the DNN models in subtype detection of RCC and grading them for prognostication.

We defined True positive (TP) as the number of cases correctly identified as RCC by the models. True negative (TN) was the number of cases correctly identified as non-RCC. False positive (FP) was the number of cases incorrectly identified as RCC and False negative (FN) was the number of cases incorrectly identified as non-RCC. Accuracy was defined as the ability to detect the presence or absence of RCC and calculated as TP + TN/TP + TN + FP + FN. Sensitivity was the ability to detect RCC cases correctly, calculated as TP/TP + FN. Specificity was the ability to detect non-RCC cases correctly, calculated as TN/TN + FP. These definitions were derived from the existing literature (15, 16). Outcomes were only recorded if the studies had reported those and were not calculated.

### 2.4 Data extraction

After removing duplicates, the retrieved articles were checked for duplicates using the EndNote 21 reference manager (17). Data was extracted using the CHARMS spreadsheet (18). All the authors extracted the data. Author information, country, total number of patients, and histopathology slides were extracted. The accuracy, sensitivity, specificity, and AUC of the models on the external dataset were collected. The author, D.C, verified the extracted data.

### 2.5 Statistical analysis

Mean ± standard deviation was used to express continuous variables, and percentages to express categorical variables. The pooled rates, mean estimates, and 95% confidence intervals (CI) were calculated using the random effects DerSimonian-Laird method (19). We used the random effects model due to the assumption that the studies were selected from a random sample and that they vary in their effect sizes (20).

Two methods evaluated heterogeneity. First, we used the Cochran Q statistic. The Cochran Q statistic tests the null hypothesis that the included studies share the same effect size. A *p*-value of <0.05 was considered significant. We then utilized the *I*^2^ statistic to detect and quantify the heterogeneity. Low, moderate, substantial, and considerable heterogeneity correspond to values <30, 31 to 60%, 61 to 75%, and > 75%, respectively, (21).

Publication bias was initially evaluated by visually examining the funnel plots and later by Egger’s test. A cut-off *p*-value of <0.05 was considered significant for the Egger’s test (22). When there was an indication of publication bias, we utilized Duval and Tweedie’s ‘Trim and Fill’ method to examine the difference in the effect size after the imputation of studies using computer software (23). The statistical analyses was conducted using the Comprehensive Meta-Analysis software, version 4 (Biostat, Englewood, NJ, USA) (24).

### 2.6 Quality assessment and risk of bias

The assessment of the individual study’s quality and risk of bias was done using the Prediction model Risk of Bias Assessment Tool (PROBAST). It contains four domains: participants, predictors, outcomes, and analysis to assess the risk of bias and applicability. A total of 20 signaling questions were used to determine if a domain was low or high risk (25). The assessment was done independently by two authors (D.C and P.S).

## 3 Results

### 3.1 Search results

**Figure 1:**
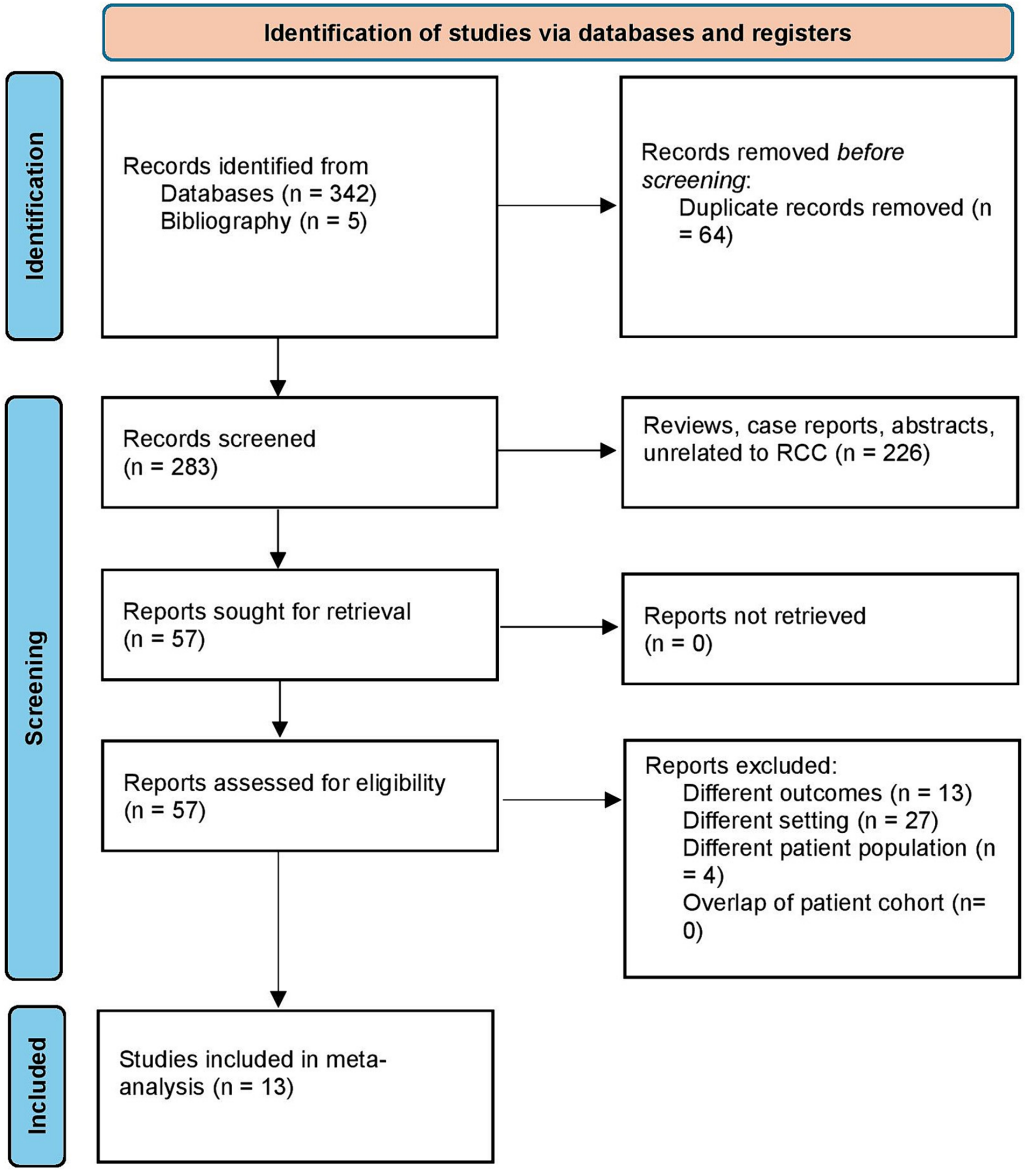
Study selection process according to the preferred reporting items for systematic reviews and meta-analysis statement.

We retrieved 347 studies using the search strategy. After the removal of duplicates, 283 studies were screened. Following this, a full text review was done on 57 studies, and finally, 13 studies were selected for the systematic review and meta-analysis (2, 26–37). The study selection process flowchart using the Preferred Reporting methods in Systematic review and Meta-analysis (PRISMA) is shown in Figure 1.

### 3.2 Study characteristics

A total of 13 studies with 13,958 slides/whole slide images from 5,340 patients were included in our analysis. There were 4 studies from the United States of America (26, 28, 29, 37), 4 from China (27, 30, 35, 36), 2 from Germany (32, 34), 1 study from the United Arab Emirates (2), India (33) and Japan (31). Eleven studies evaluated the performance of CNN models (2, 26–31, 33–35, 37). Schulz et al. (32) assessed the performance of a multimodal deep learning model (MMDLM). Zheng et al. (36) evaluated a deep learning model based on a clustering-constrained-attention multiple-instance learning (CLAM) framework called SSL-CLAM. The DNN models were used to subtype RCC in 8 studies (2, 26–29, 33, 35, 37). Survival prediction and prognosis was assessed by 5 studies (30–32, 34, 36). The Cancer Genome Atlas (TGCA) validated the machine learning model in 12 studies (2, 26, 28–37). Two studies also used the Clinical Proteomic Tumor Analysis Consortium (CPTAC) together with the TGCA dataset to validate their model (29, 36). The study characteristics and the DNN details are summarized in Tables 1, 2.

**Table 1:** Summary of the included studies.

Study, year	Country	Total number of patients, *n*	Histopathology evaluated	External dataset used for validation after internal validation	Outcome assessed
Abdeltawab et al. (2022) (26)	USA	41	Clear cell RCC and papillary RCC	TCGA	RCC subtype
Cai et al. (2022) (27)	China	243	All subtypes of RCC and normal histopathology	None	RCC subtype or healthy kidneys
Fenstermaker et al. (2020) (28)	USA	42	All subtypes of RCC and normal histopathology	TCGA	RCC subtype or healthy kidneys and Fuhrman grade
Abu Haeyeh et al. (2022) (2)	UAE	52	All subtypes of RCC	None	RCC subtype
Marostica et al. (2021) (29)	USA	1,150	All subtypes of RCC	TCGA, CPTAC	RCC subtype, survival prediction and genetic profiles.
Ning et al. (2020) (30)	China	209	Clear cell RCC	TCGA	Prediction of survival of clear cell RCC
Ohe et al. (2022) (31)	Japan	530	Clear cell RCC	TCGA	Classification of clear cell RCC into clear and eosinophilic phenotypes
Schulz et al. (2021) (32)	Germany	248	Clear cell RCC	TCGA	Disease specific survival and 5-year survival in patients with clear cell RCC
Tabibu et al. (2019) (33)	India	1,584	All subtypes of RCC	TCGA	RCC subtype
Wessels et al. (2022) (34)	Germany	353	Clear cell RCC	TCGA	Prediction of 5-year overall survival in patients with clear cell RCC
Wu et al. (2021) (35)	China	153	All subtypes of RCC	TCGA	RCC subtype
Zheng et al. (2023) (36)	China	735	Clear cell RCC	TCGA, CPTAC	Grade clear cell RCC per WHO-ISUP
Zhu et al. (2021) (37)	USA	Not reported	All subtypes of RCC	TCGA	RCC subtype

CAPTAC, Clinical Proteomic Tumor Analysis Consortium; RCC, renal cell carcinoma; TCGA, The Cancer Genome Atlas.

**Table 2:** Deep neural network model characteristics.

Study	Machine learning type	Model architecture	Feature extraction and training process using images	Outcome
Abdeltawab et al. (26)	CNN	Developed using Tensor-Flow from Google, the performance was compared with pretrained ResNet18 and ResNet34.	Whole slide images were divided into smaller patches; 250 × 250, 350 × 350 and 450 × 450 pixels. Training set included 44 WSI and final validation included 20 WSI.	The framework was able to subclassify into fat, parenchyma, clear cell renal cell carcinoma, and clear cell papillary renal cell carcinoma. It showed better performance compared to ResNet and other deep learning methods.
Cai et al. (27)	CNN	Based on Alex-Net	Whole slide images of 93 patients with renal cancer and 150 healthy people were used. Feature vectors were collected and fused for training.	Model performed well in classifying RCC. Combination of both deep learning with texture descriptors resulted in increased renal cancer detection accuracy.
Fenstermaker et al. (28)	CNN	The model consisted of 6 convolutional layers and 6 other layers.	Samples from 42 patients were obtained from TCGA. Slides were divided into 1,024 × 1,024 pixels patches. The model trained over the dataset around 25 times.	The model showed 97.5% accuracy in distinguishing clear cell, papillary, and chromophobe subtypes and a 98.4% accuracy in predicting Fuhrman grade.
Abu Haeyeh et al. (2)	CNN	Based on ResNet 50	A weakly supervised model was created and pre annotated WSI by pathologists was used in training. Multiple instance learning with overlapping patches was employed.	The framework was able to achieve 93% accuracy and outperformed ResNet-50.
Marostica et al. (29)	CNN	Constructed using VGG-16, Inception v3 and ResNet-50	A total of 2,363 WSI were used from TCGA and 782 WSI from CPTAC cohort. A weakly supervised approach was used and the three different CNNs were compared.	The model was able to identify RCC subtype, survival and identify correlations between genetic aberrations and histology.
Ning et al. (30)	CNN	Developed with several blocks and fully connected layers and global pooling applied in the end	The CNN was trained using an average of 150 patches, each with a size of 128×128 pixels. The data was later combined with functional genomic data to identify high risk groups.	The model was effective in predicting the prognosis of clear cell RCC. The study also evaluated the correlation between renal cancer and genetic data.
Ohe et al. (31)	CNN	Based on AlexNet	Clear and eosinophilic regions of 227 × 227 pixels were obtained from 3,904 and 16,584 regions.	The model detected clear and eosinophilic regions with high accuracy. It also predicted outcomes using histopathological and gene signatures.
Schulz et al. (32)	MMDLM	Constructed using 18 layers of ResNet.	About 230 WSI were used in unimodal training, and later multimodal training was done. CT and MRI from the same cohort was also used.	The model was able to predict the 5-year survival status and the accuracy increased when combined with radiological and genomic data.
Tabibu et al. (34)	CNN	Developed using modification of the pre-trained ResNet 18 and ResNet 34.	Images extracted were made into patches of size 512 × 512 pixels and data was augmented. The average number of epochs were between 3 and 40.	The model was able to diagnose RCC and distinguish between the subtypes. Prediction of high-risk types was also accomplished.
Wessels et al. (34)	CNN	Based on ResNet 18.	The CNN was trained in two stages using 254 pixels WSI and patches were augmented.	The model was able to predict the 5 year overall survival with an AUROC of 0.78. The accuracy increased when the CNN prediction was combined with other data such as age, tumor size and metastasis.
Wu et al. (35)	CNN	Based on Inception V3.	From the images annotated by pathologists, each subtype of RCC was entered into the training dataset. The size of the regions included was 512 × 512 pixels.	The model was able to subtype the RCC as well as grade them per WHO-ISUP.
Zheng et al. (36)	CLAM	The model used was SSL-CLAM, a weakly supervised deep learning method.	The model was trained using 519 WSI from TCGA and 783 WSI from CPTAC datasets. The patches extracted from WSI were 256 × 256 pixels.	The model was able to successfully designate a Fuhrman grading.
Zhu et al. (37)	CNN	Based on ResNet	Two pathologists annotated 486 WSI. Patches extracted from WSI were 224×224 pixels and used in training the model over 40 epochs.	The model performed well in subtyping RCC

CLAM, clustering-constrained-attention multiple-instance learning; CNN, convolutional neural networks; CT, Computed tomography; MMDLM, Multimodal deep learning model; MRI, Magnetic resonance imaging; WHO-ISUP, World health organization-International society of urologic pathologists; WSI, Whole slide images.

### 3.3 Outcomes

**Figure 2:**
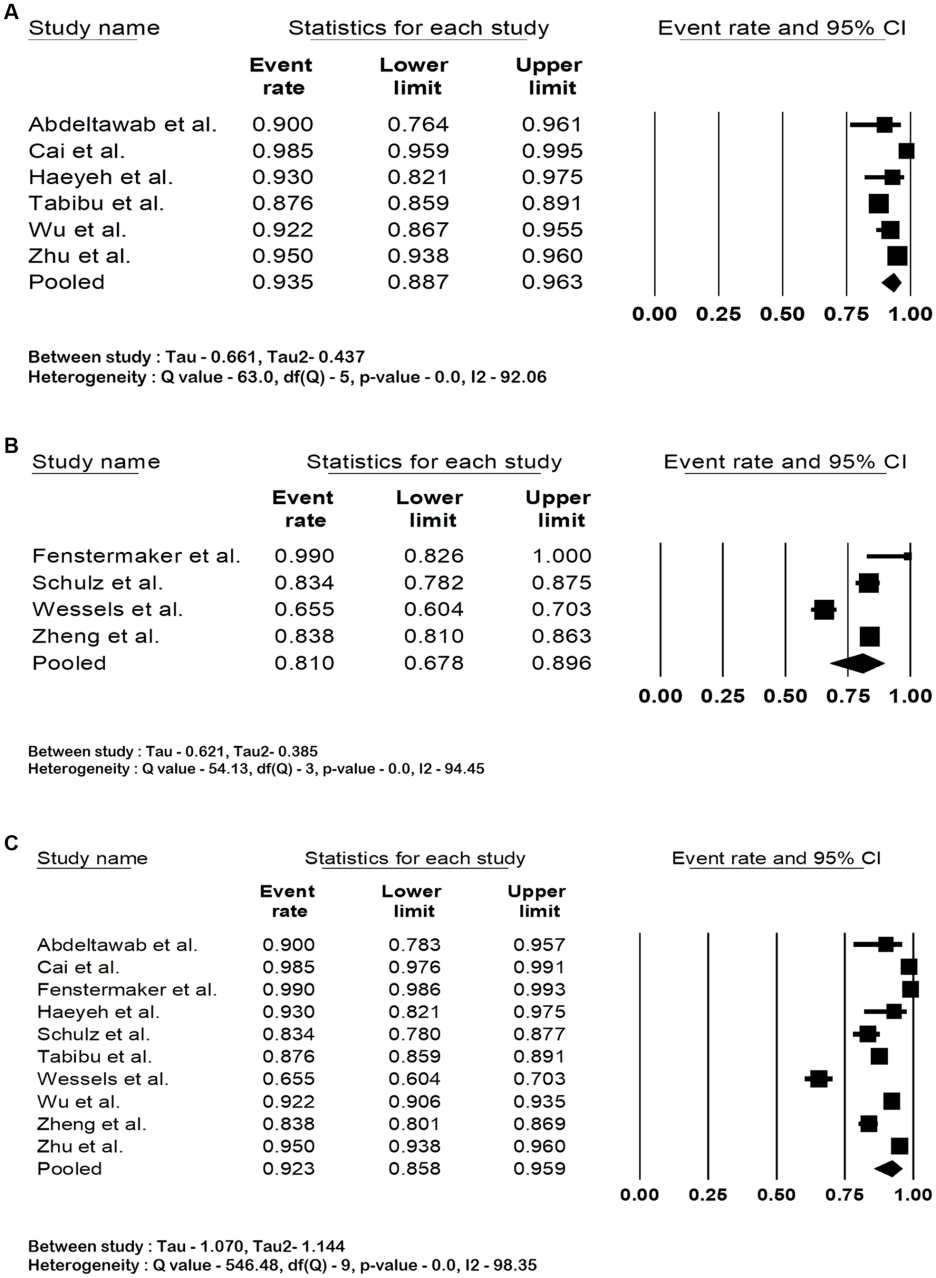
Forest plots showing **(A)** accuracy in renal cell carcinoma subtype detection, **(B)** accuracy in renal cell carcinoma survival analysis **(C)** overall accuracy of deep neural network in detection and prognostication of renal cell carcinoma.

**Figure 3:**
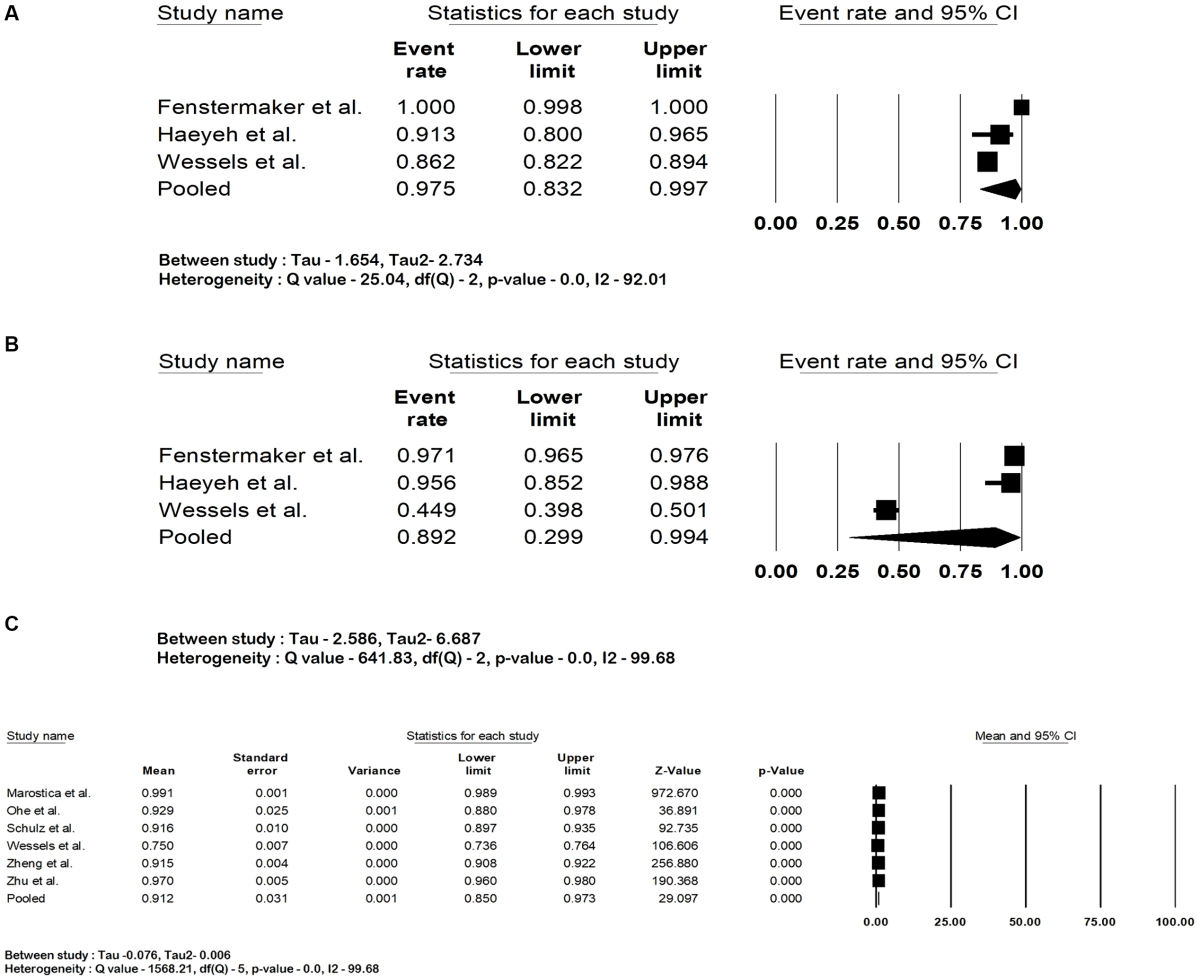
Forest plots showing **(A)** sensitivity, **(B)** specificity and **(C)** area under the curve of deep neural network in detection and prognostication of renal cell carcinoma.

The pooled accuracy of the DNN in the detection of RCC subtype was 93.5% (95% CI: 88.7–96.3; *I*^2^ = 92%). The pooled accuracy in survival analysis was 81% (95% CI: 67.8–89.6; *I*^2^ = 94.4%). They had an overall accuracy of 92.3% (95% CI: 85.8–95.9; *I*^2^ = 98.3%) when used for RCC detection and survival analysis. The forest plots are shown in Figures 2A–C. The studies pooled together had a sensitivity of 97.5% (95% CI: 83.2–99.7; *I*^2^ = 92%), specificity of 89.2% (95% CI: 29.9–99.4; *I*^2^ = 99.6%) and area under the curve of 0.91 (95% CI: 0.85–0.97.3; *I*^2^ = 99.6%). The forest plots are shown in Figures 3A–C.

### 3.4 Quality assessment and risk of bias

**Figure 4:**
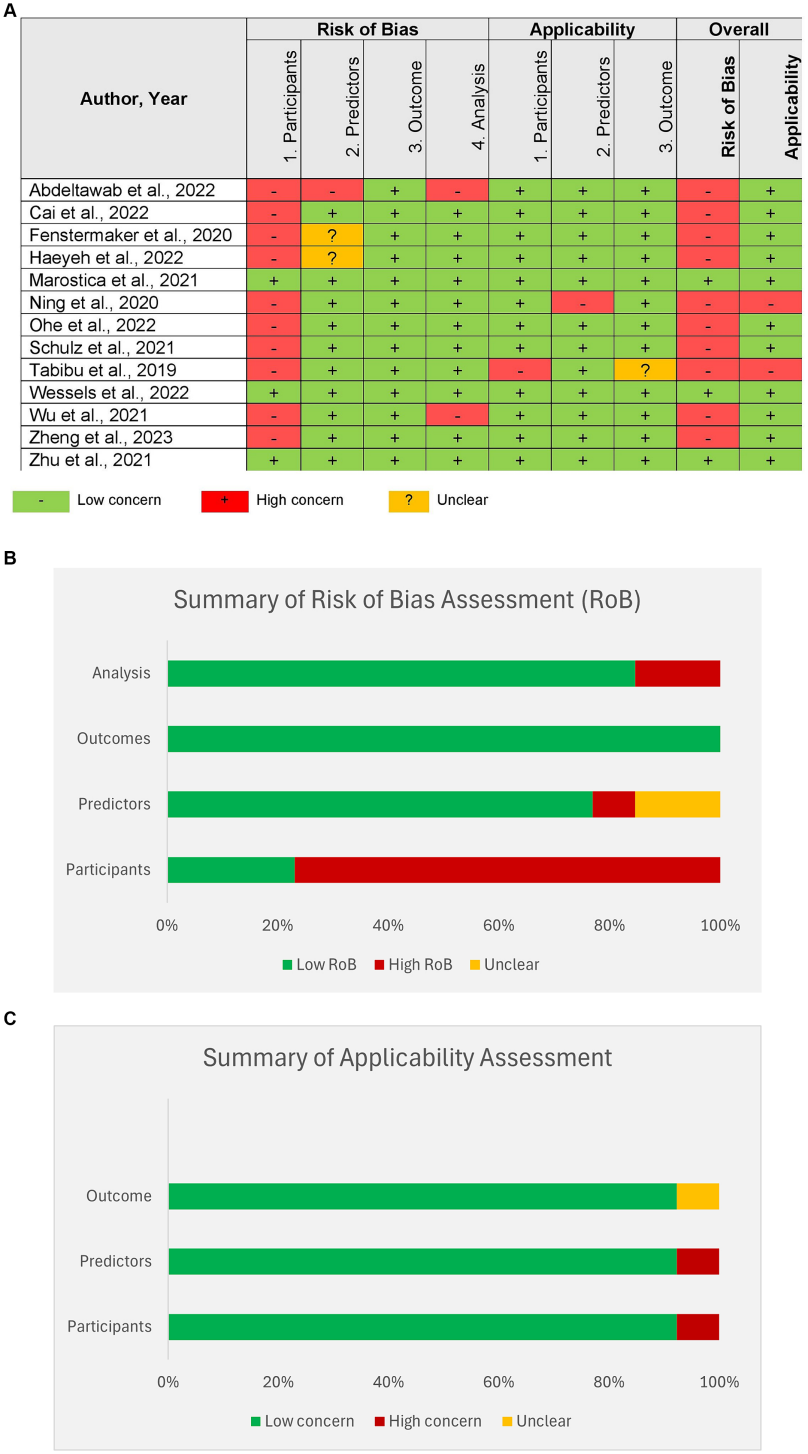
Risk of bias assessment of studies by Prediction model Risk of Bias Assessment Tool (PROBAST). **(A)** Assessment of individual studies, **(B)** summary of Risk of bias assessment for all studies, **(C)** summary of applicability for all studies.

Most of the studies showed a high risk of bias in the selection of study participants. Figure 4A shows the results of the PROBAST scoring of individual studies. Figures 4B,C show the summary of the risk of bias and applicability across all studies.

### 3.5 Heterogeneity

Both the Q statistic and I2 statistics were utilized to assess heterogeneity. Upon quantification of the heterogeneity, we concluded that the degree of heterogeneity was considerable, as they exceeded 75%.

### 3.6 Sensitivity analysis

Sensitivity analysis was performed by eliminating one study at a time to determine whether there is any difference in the effect sizes. We found no significant differences except in the analysis of pooled specificity. This was due to the reported specificity of 44.9% by Wessels et al., which was lower than other studies. The sensitivity analysis of all the outcomes is shown in the Supplementary material.

### 3.7 Publication bias

**Figure 5:**
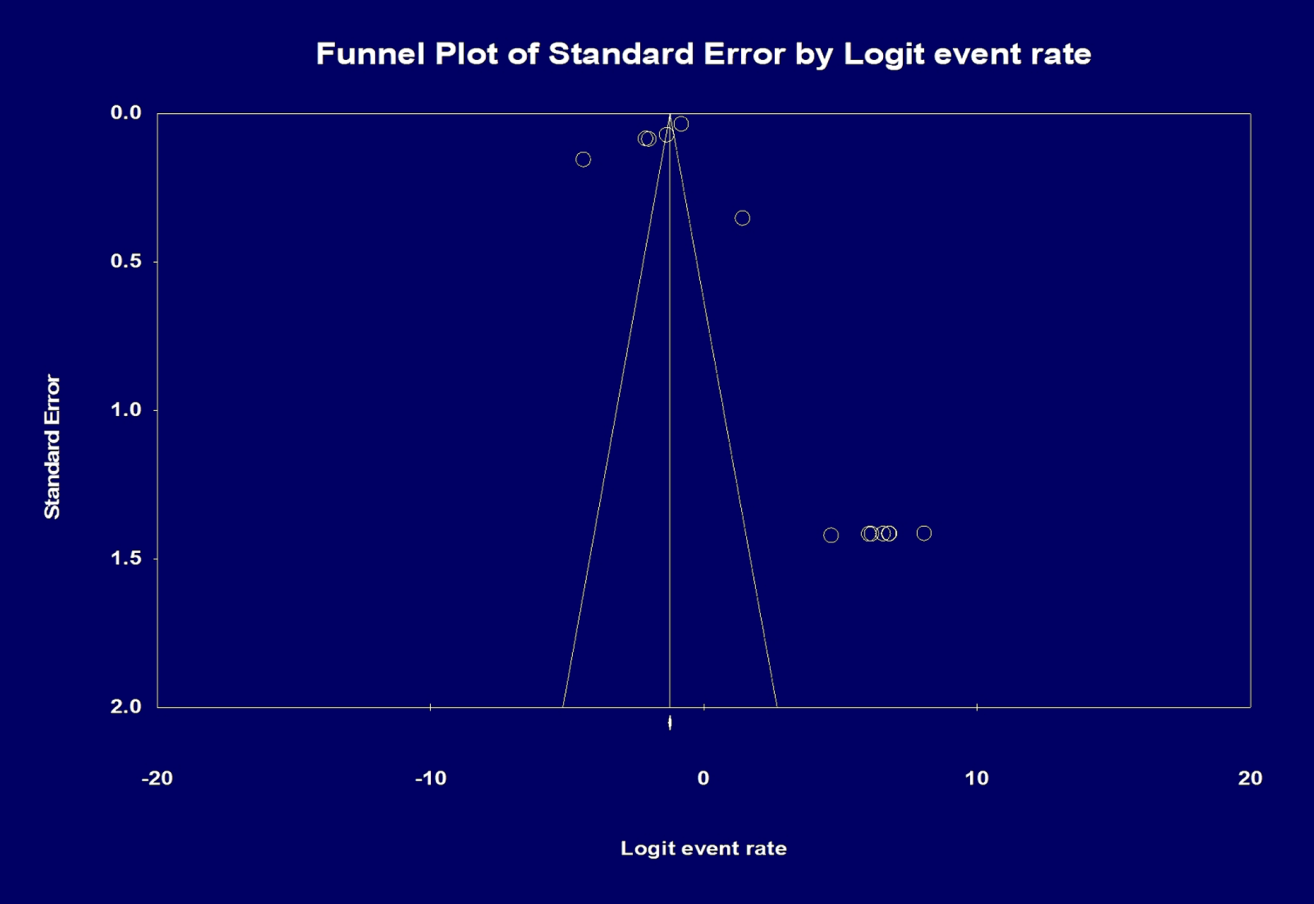
Analysis of publication bias by funnel plot showing the effect size of the total number of patients and the total number of histopathology slides/whole slide images. Egger’s test for a regression intercept gave a 1-tailed *p*-value of 0.284 indicating no publication bias. The intercept (B0) is 1.942, 95% confidence interval (−5.326 and 9.211), with *t* = 0.588, df = 11.

Analysis of Publication Bias was done initially by visual inspection, and it showed a potential publication bias due to the presence of asymmetry. Therefore, an Egger’s test was performed, and the regression intercept gave a 1-tailed *p*-value of 0.28, indicating the lack of publication bias. The funnel plot with the observed and imputed studies is shown in Figure 5.

## 4 Discussion

Our systematic review and meta-analysis demonstrate that deep machine learning can be utilized to diagnose renal cell carcinoma, classify subtypes, and grade RCC. Based on our analysis, the DNN models had excellent performance. The pooled accuracy was 92.3%, sensitivity was 97.5%, specificity was 89.2%, and area under the curve 0.91.

Artificial intelligence (AI) in pathology or computational pathology, referred to as pathomics, is a rapidly developing field. Whole-slide imaging (WSI) technology has allowed the capture and storage of histopathologic images into a high-resolution virtual slide, which is used to train deep learning algorithms (38).

At present, deep learning methods are the most successful among other machine learning types in detecting abnormalities in histopathologic images (27). CNNs, by their design, can detect spatial information and compare images (39). These can then be used for deep feature extraction in a weakly supervised or unsupervised learning setting to identify relationships between random variables in a large dataset (36). A supervised approach is where the WSI have annotations showing the irregularity in histopathology, which the machine learning model then uses as a representative to learn from (2). Similarly, the MMDLM uses clinical, radiologic, and histopathological data to train its algorithm and a “fusion” approach to reach a conclusion. Schulz et al. used MMDLM to predict the prognosis and survival among patients with ccRCC (32). Big data is essential to develop and train such deep learning algorithms. In the field of renal malignancies, the TCGA dataset is an excellent resource for genetic, pathologic, molecular, and clinical data that could be used to train and validate these models (1).

Various architecture frameworks have been used to construct a CNN model. These networks comprise several interconnected layers composed of several blocks (30). One of the more commonly used architectures is the ResNet (residual network), which allows more deeper layers to be created and reduces errors (39). ResNet architecture based CNN has been found to have better performance than the Inception-v3 and VGG-16 (visual geometry group) (29).

Typically, in oncology, clinical decision-making involves multiple data points such as biomarkers, gene expression profiling, and radiology imaging. Machine learning algorithms can help in combining various data to improve detection. Eigengenes extraction and radiomics, where CNN can extract genetic and radiology information to augment the prediction accuracy has good outcomes (30). The relationship between copy number alterations (CNAs), a common cause of gene alterations in malignancies, and histopathology can also be elucidated using machine learning. Marostica et al. demonstrated that their model recognized histopathological changes in CNAs involving VHL (von Hippel–Lindau), EGFR (epidermal growth factor), and KRAS (Kristen rat sarcoma virus) genes. Their model also distinguished between low and high-risk RCC and predicted overall survival (29).

Another study by Ning et al. used a combination of features extracted from computed tomography (CT) and histopathology added to eigengenes to create a prognostic model for ccRCC (30).

A high percentage of patients with RCC face recurrence after surgical resection, and current predictive models lack the ability to predict recurrence accurately. DNNs can assist in prognostication and determine survival (30, 32, 40). The model used by Wessels et al. was able to predict the 5-year overall survival (OS) with an AUC of 0.78. The model’s accuracy increased when other data points, such as age, tumor size, and metastasis were added (34). Ohe et al. (31) used their CNN model based on AlexNet to grade ccRCC into clear and eosinophilic types according to the WHO/ISUP system to predict prognosis. When evaluating survival analysis, the concordance index (C-Index) is used to determine the efficacy of matching patients according to their risk. The studies by Ning, Ohe and Sculz et al. reported good performance of their model’s C-index (30–32).

More recently, a study by Chen et al. demonstrated that assessing various cancer types was possible through a self-supervised learning model. The model, called UNI, a Vision Transformer (ViT) based model, could pretrain using more than 100 million images from different datasets and evaluate 34 different histopathologies of varying difficulties. Its performance was superior, particularly while assessing ccRCC and prostate adenocarcinoma histopathologies. The ability to integrate different datasets and perform large quantities of tasks demonstrates that such models could be utilized in the near future to complete large-scale histopathological tasks without compromising diagnostic accuracies (41).

Our study has some limitations. First, all the studies were retrospective, and the data depended on the accuracy of the collection process. Second, there is also a possibility for the introduction of selection bias when datasets were accessed to include patients with RCC or a particular subtype of RCC. Third, although most of the models included in the study were CNN-based, differences exist in the structure and construct of these models. Lastly, heterogeneity was noted in our analyses due to these differences in the models. Therefore, caution must be observed while interpreting these results.

To our knowledge, this is the first meta-analysis to assess the performance of machine learning models in the diagnosis, subtyping and prognostication of RCC using histopathology. Histopathologic classification of renal cell carcinoma into its subtypes and grading is a challenging task. Deep learning can help fill a large void in the early detection of RCC as well as accurate determination of its subtypes. Although it cannot replace the skill and experience of a pathologist or radiologist, it can decrease their workload and improve efficiency.

## Data Availability

The original contributions presented in the study are included in the article/Supplementary material, further inquiries can be directed to the corresponding author.
